# Temperature-dependent optical and vibrational properties of PtSe_2_ thin films

**DOI:** 10.1038/s41598-020-76036-y

**Published:** 2020-11-04

**Authors:** Desman P. Gulo, Han Yeh, Wen-Hao Chang, Hsiang-Lin Liu

**Affiliations:** 1 Department of Physics, National Taiwan Normal University, Taipei, 11677 Taiwan; 2 Department of Electrophysics, National Chiao Tung University, Hsinchu, 30010 Taiwan

**Keywords:** Materials science, Nanoscience and technology, Optics and photonics

## Abstract

PtSe_2_ has received substantial research attention because of its intriguing physical properties and potential practical applications. In this paper, we investigated the optical properties of bilayer and multilayer PtSe_2_ thin films through spectroscopic ellipsometry over a spectral range of 0.73–6.42 eV and at temperatures between 4.5 and 500 K. At room temperature, the spectra of refractive index exhibited several anomalous dispersion features below 1000 nm and approached a constant value in the near-infrared frequency range. The thermo-optic coefficients of bilayer and multilayer PtSe_2_ thin films were (4.31 ± 0.04) × 10^−4^/K and (–9.20 ± 0.03) × 10^−4^/K at a wavelength of 1200 nm. Analysis of the optical absorption spectrum at room temperature confirmed that bilayer PtSe_2_ thin films had an indirect band gap of approximately 0.75 ± 0.01 eV, whereas multilayer PtSe_2_ thin films exhibited semimetal behavior. The band gap of bilayer PtSe_2_ thin films increased to 0.83 ± 0.01 eV at 4.5 K because of the suppression of electron–phonon interactions. Furthermore, the frequency shifts of Raman-active *E*_*g*_ and *A*_*1g*_ phonon modes of both thin films in the temperature range between 10 and 500 K accorded with the predictions of the anharmonic model. These results provide basic information for the technological development of PtSe_2_-based optoelectronic and photonic devices at various temperatures.

## Introduction

Two-dimensional transition-metal dichalcogenides (TMDs) have attracted considerable attention because of their novel physical properties in the reduced dimension and potential practical applications^1–5^. TMDs (common formula MX_2_ [M = Mo, W; X = S, Se]) possess substantial band gap energies (approximately 1.0 to 2.0 eV) because of their quantum confinement effect and weak van der Waals forces^3,6–10^. Subsequent studies^11–13^ have revealed that these characterizations can be altered simply by tuning their thickness. Furthermore, indirect to direct band gap transitions occur when the thickness is reduced to a monolayer. Additionally, TMDs exhibit high carrier mobility and high quantum efficiency of photoluminescence yield^12,14,15^. These superior properties make TMDs very attractive materials for use in optoelectronics^16^, photovoltaic^17^, and field-effect transistors^18^.

PtSe_2_ has received much research interest as an emerging TMDs material^19^. It exhibits unique thickness-dependent type-II Dirac semimetal to semiconducting transition^20,21^. Moreover, PtSe_2_ exhibits strong interlayer coupling and excellent carrier mobility^22^. It is a promising material for use in next-generation sensors, optoelectronics, and ultrafast photonic devices^23–25^. Circular polarization calculations have revealed that single-layer PtSe_2_ exhibits strong circular dichroism polarization along the M–K direction and near the Γ point. Thus, PtSe_2_ is an excellent candidate for valleytronic devices^26^. In light of the many potential applications of PtSe_2_, a comprehensive study of optical properties for PtSe_2_ is essential. The temperature-dependent optical constants of PtSe_2_ are critical references for determining the effects of self-heating on a device. O’Brien et al*.*^27^ studied the Raman scattering spectra of direct-selenization grown PtSe_2_ thin films as a function of film thickness, laser wavelength, and laser polarization. They found that the positions of phonon modes of PtSe_2_ thin films exhibited a redshift with increase in the thickness from 0.5 to 5.0 nm. This phenomenon was attributed to domination of stacking-induced structural changes and Columbic interaction effects when the number of layers of the PtSe_2_ thin films was increased. Yu et al*.*^28^ reported that PtSe_2_ thin films possess variable band gaps in the mid-infrared frequency region. They showed that monolayer PtSe_2_ is suitable for use in visible and near-infrared photodetectors. Thus, bilayer PtSe_2_ thin films are compatible with mid-infrared photodetectors and are an excellent candidate for photoelectronic devices. Xie et al*.*^29^ investigated the room-temperature optical constants of PtSe_2_ for different thicknesses through spectroscopic ellipsometry. They found that the values of refractive index and extinction coefficient exhibited a strong dependence on thickness.

Most optical measurements of PtSe_2_ thin films have been limited to room temperature^27–29^. The temperature-dependent optical properties of PtSe_2_ thin films have not been reported. In this paper, we characterized the optical constants of PtSe_2_ thin films over a wide range of photon energy (from 0.73 to 6.42 eV) and temperature (between 4.5 and 500 K) through spectroscopic ellipsometry. We found that bilayer PtSe_2_ thin films exhibited an indirect band gap and that multilayer PtSe_2_ thin films exhibited semimetal behavior at 300 K. Furthermore, the band gap of bilayer PtSe_2_ thin films increased from 0.71 ± 0.01 to 0.83 ± 0.01 eV when temperature decreased from 500 to 4.5 K. Multilayer PtSe_2_ thin films exhibited a band gap of approximately 0.04 ± 0.004 eV at 500 K. Additionally, we investigated the temperature-dependent Raman scattering spectra of PtSe_2_ thin films from 10 to 500 K. The temperature dependence of *E*_*g*_ and *A*_*1g*_ phonon modes was attributed to the anharmonic contributions to the interatomic potential energy, mediated by phonon–phonon interactions. These findings are highly promising for further development of PtSe_2_ thin films in optoelectronic and photonic applications at various temperatures.

## Experiment

**Figure 1:**
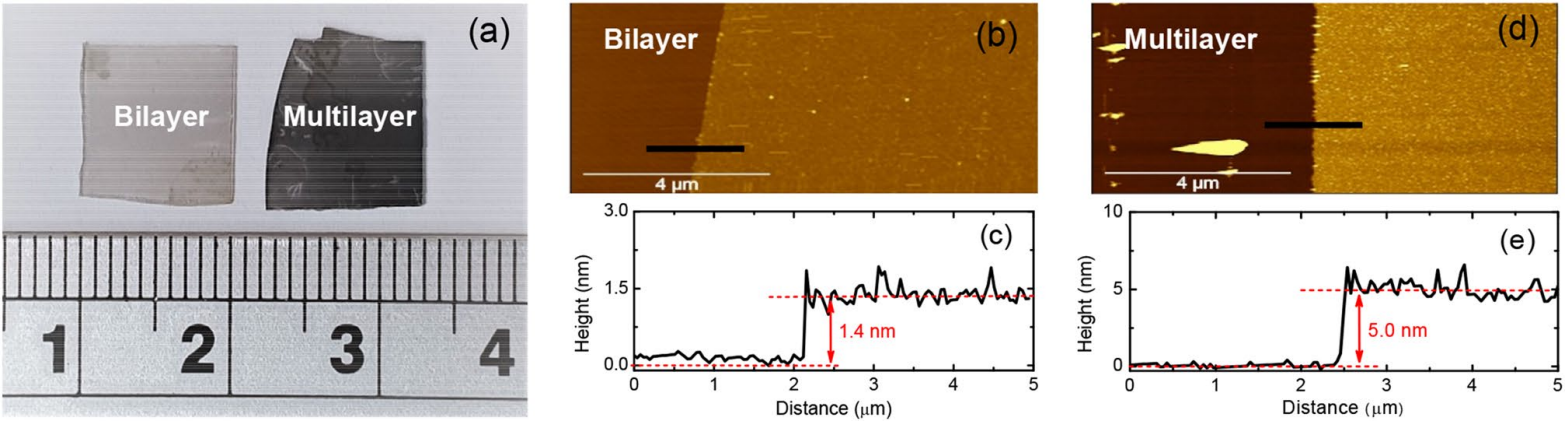
(**a**) Optical microscopic images of two PtSe_2_ thin films. (**b**–**e**) Atomic force microscopic images and corresponding height profiles of two PtSe_2_ thin films.

Bilayer and multilayer PtSe_2_ thin films were grown on sapphire substrates by using chemical vapor deposition method. Platinum (II) chloride (PtCl_2_) and selenium (Se) were used as precursors. Both PtSe_2_ samples with an area of approximately 1 cm^2^ were formed at a temperature of 400 °C. The growth time ranged from 10 to 20 min for bilayer and multilayer thin films, respectively. The samples were prepared according to the method described in^21^. Figure 1 depicts the optical microscopic and atomic force microscopic images, and corresponding height profiles of two thin films. The thickness of these two thin films was approximately 1.4 and 5.0 nm, respectively, thus corresponding to two and seven PtSe_2_ layers. Both thin films were also verified through transmission electron microscopy to be high-quality samples^30^.

Room-temperature spectroscopic ellipsometry spectra were recorded under incident angles of 60°, 65°, 70°, and 75° and over a spectral range of 0.73 to 6.42 eV by using an ellipsometer (J. A. Woollam Co. M-2000U). For temperature-dependent measurements between 4.5 and 500 K, the samples were placed in a Janis ST-400 ultrahigh-vacuum continuous-flow helium cryostat^31,32^. The optical constants were obtained through spectroscopic ellipsometry using the stacked layer model (sapphire substrate/thin film/surface roughness/air ambient structure)^32^. The parameters of the model used to fit the raw ellipsometry data are listed in Table 1. The values of the mean square error are 1.07 and 1.40 for bilayer and multilayer PtSe_2_ thin films. The independently measured experimental data at different incident angles and modeled curves exhibited good agreement (see Supplementary Figs. 1 and 2).

**Table 1:** Parameters of the stacked layer model fit for bilayer and multilayer PtSe_2_ thin films.

Layer type	Bilayer	Multilayer
Sapphire substrate (mm)	1	1
Film (nm)	1.45 ± 0.3	5.10 ± 0.6
Surface roughness (nm)	0.10 ± 0.02	0.25 ± 0.03

**Figure 2:**
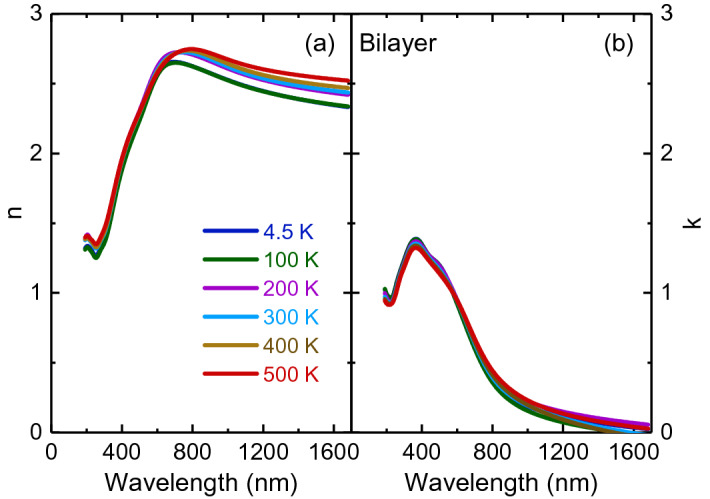
Temperature-dependent (**a**) refractive index and (**b**) extinction coefficient of bilayer PtSe_2_ thin film.

Room-temperature micro-Raman scattering measurements were performed using a backscattering geometry with a laser excitation wavelength of 532 nm^31^. The linear polarized light was focused to a 3-μm-diameter spot on the sample surface. Subsequently, a SENTERRA spectrometer collected and dispersed the scattered light with a 1024-pixel-wide charge-coupled detector. The spectral resolution achieved using these instruments was generally less than 0.5 cm^−1^. To avoid heating effects, the laser power was set to less than 0.5 mW. The sapphire substrate exhibited high thermal conductivity and evacuated heating well^33^. The samples were mounted in a continuous-flow helium cryostat and LINKAM heating stage, which enabled measurements in the temperature range of 10–500 K^31^.

## Results and discussion

**Figure 3:**
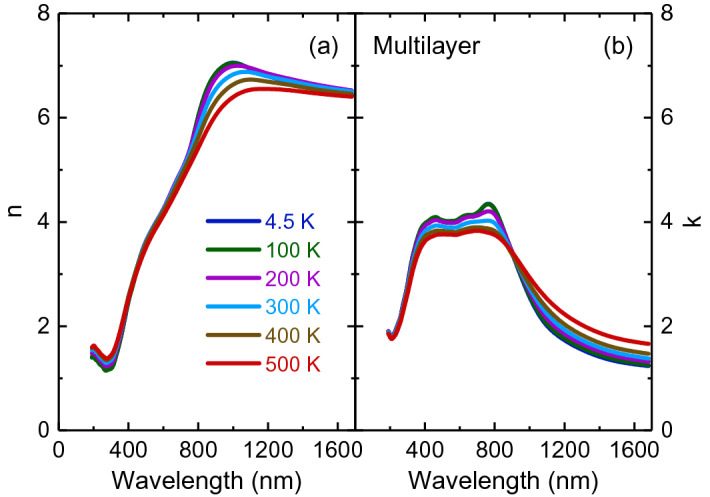
Temperature-dependent (**a**) refractive index and (**b**) extinction coefficient of multilayer PtSe_2_ thin film.

Figures 2 and 3 illustrate the temperature-dependent optical constant spectra of bilayer and multilayer PtSe_2_ thin films recorded in a wavelength range of 193 to 1700 nm obtained through spectroscopic ellipsometry analysis. The spectra were almost identical by rotating the sample’s azimuthal orientation of 45° and 90° shown in the Supplementary Figs. 3 and 4, indicating the in-plane isotropic optical properties of PtSe_2_ thin films. For both thin films, the room-temperature refractive index increased substantially with an increase in the wavelength in the spectral range from 193 to 700 nm for bilayer and from 193 to 1100 nm for multilayer thin films. It then approached one maximum, which corresponded with the anomalous dispersion regime^34^ and finally decreased with the wavelength until the wavelength reached 1700 nm. With an increase in layer thickness, the value of refractive index increased. This phenomenon is consistent with previous reports of thickness-dependent optical constants^29^. Notably, multilayer PtSe_2_ thin film exhibited large refractive index values (approximately 6.88 at a wavelength of 1070 nm). Bilayer PtSe_2_ thin film exhibited a maximum refractive index of approximately 2.72 at a wavelength of 720 nm. The characteristics of high refractive indices of bilayer and multilayer PtSe_2_ thin films indicated a large scattering cross-section inside the crystal^35^. Thus, these effects are highly favorable for light trapping in photonic^36^ and optoelectronic^37^ devices in the visible to near-infrared frequency range. With a decrease in temperature, the refractive index of bilayer PtSe_2_ thin film decreased in the near-infrared region. This behavior is ascribed to the decreased electron–phonon interaction with a decrease in temperature, as observed for other semiconducting TMDs, such as monolayer MoS_2_, MoSe_2_, WS_2_, and WSe_2_^32^. By contrast, this trend was reversed for multilayer PtSe_2_ thin film, which could be associated with the enhanced electron–phonon interaction. The thermo-optic coefficients (∂n/∂T) for bilayer and multilayer PtSe_2_ thin films were (4.31 ± 0.04) × 10^−4^/K and (− 9.20 ± 0.03) × 10^−4^/K, respectively, at a wavelength of 1200 nm. Bilayer PtSe_2_ thin film exhibited a positive thermo-optic coefficient value, similar to the coefficient of other semiconducting materials such as silicon^38^. By contrast, multilayer PtSe_2_ thin film had a negative value of thermo-optic coefficient that was similar to the coefficient of TiO_2_^39^. The negative thermo-optic coefficient observed in multilayer PtSe_2_ may arise from the enhanced electron–phonon interaction at low temperatures because of its semimetallic properties. The extinction coefficient spectra (Figs. 2 and 3) of two thin films were featureless above 1000 nm, but exhibited several absorption peaks below 1000 nm. These absorptions exhibited a blueshift trend with a decrease in temperature. A detailed analysis of temperature-dependent optical absorption is provided later.

**Figure 4:**
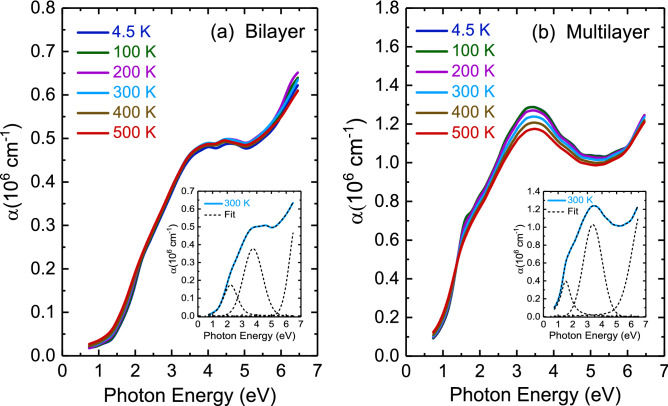
Temperature-dependent optical absorption spectra of (**a**) bilayer and (**b**) multilayer PtSe_2_ thin films. The inset illustrates the best fit using the Lorentz-Gaussian function at 300 K.

**Figure 5:**
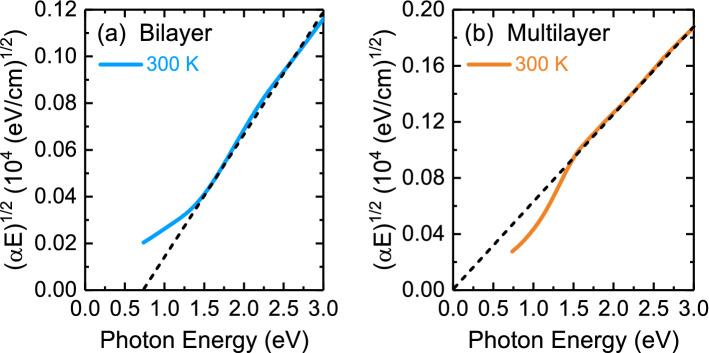
The plot of (α·*E*)^1/2^ vs. photon energy enables the extraction of indirect band gap of (**a**) bilayer and (**b**) multilayer PtSe_2_ thin films at 300 K.

Figure 4 presents the temperature-dependent optical absorption coefficient spectra of two thin films. The absorption spectra were fitted using the Lorentzian-Gaussian function, as illustrated by the dashed lines in the inset of Fig. 4. Bilayer PtSe_2_ thin film exhibited two optical absorption bands at approximately 2.22 and 3.75 eV. Multilayer PtSe_2_ thin film revealed two optical absorption bands at approximately 1.51 and 3.39 eV. In accordance with previous reports^20,40^, all absorption peaks for two thin films were assigned to charge-transfer excitations between the hybridization of Pt 5*d* and Se 4*p* states at the valence and conduction band edges. Moreover, the first-principles calculations^20^ predicted that the rapid decrease in band energy of conduction band states and increase in valence band states lead to metallization starting from trilayer PtSe_2_ thin film. As a result, two absorption bands observed in multilayer PtSe_2_ thin film exhibited a redshift as compared to those of bilayer PtSe_2_ thin film. The band gap energy of bilayer and multilayer PtSe_2_ thin films is depicted Fig. 5. The optical absorption coefficient, which includes contributions from both direct and indirect band gap transitions, was analyzed using the following expression^41^:

1 $$ {\upalpha }\left( E \right) = \frac{A}{E}\left( {{\text{E}} - E_{g,dir} } \right)^{0.5} + \frac{B}{E}\left( {E - E_{g,ind} \mp E_{ph} } \right)^{2} $$

where *E*_*g,dir*_ and *E*_*g,ind*_ are the magnitudes of direct and indirect band gaps, respectively; *E*_*ph*_ is the emitted (absorbed) phonon energy, and *A* and *B* are constants. This model, which assumes a simple band shape, enables extraction of the direct band gap when (α·*E*)^2^ is plotted as a function of photon energy and extraction of indirect band gap when (α·*E*)^1/2^ is plotted as a function of photon energy. Figure 5 presents an indirect band gap of approximately 0.75 ± 0.01 eV at 300 K for bilayer PtSe_2_ thin film, whereas multilayer PtSe_2_ thin film exhibited semimetal behavior from 300 to 4.5 K (see Supplementary Fig. 5). Plotting (α·*E*)^2^ as a function of photon energy for both thin films led to a negative value for the band gap. The results of our band gap analysis of bilayer PtSe_2_ thin film were consistent with those of other experimental studies^29,41,42^. However, the results for multilayer PtSe_2_ thin film differed from those of other reports of semiconducting behavior in films of a similar thickness^29,42,43^. One possibility that could account for these differences is due to different quality of thin films.

**Figure 6:**
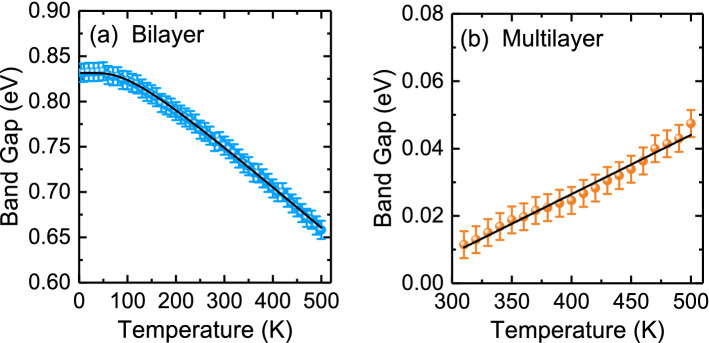
(**a**) Temperature-dependent indirect band gap of (**a**) bilayer and (**b**) multilayer PtSe_2_ thin films. The thin solid lines indicate the results of the fitting using (**a**) the Bose–Einstein model and (**b**) the linear fit model.

**Figure 7:**
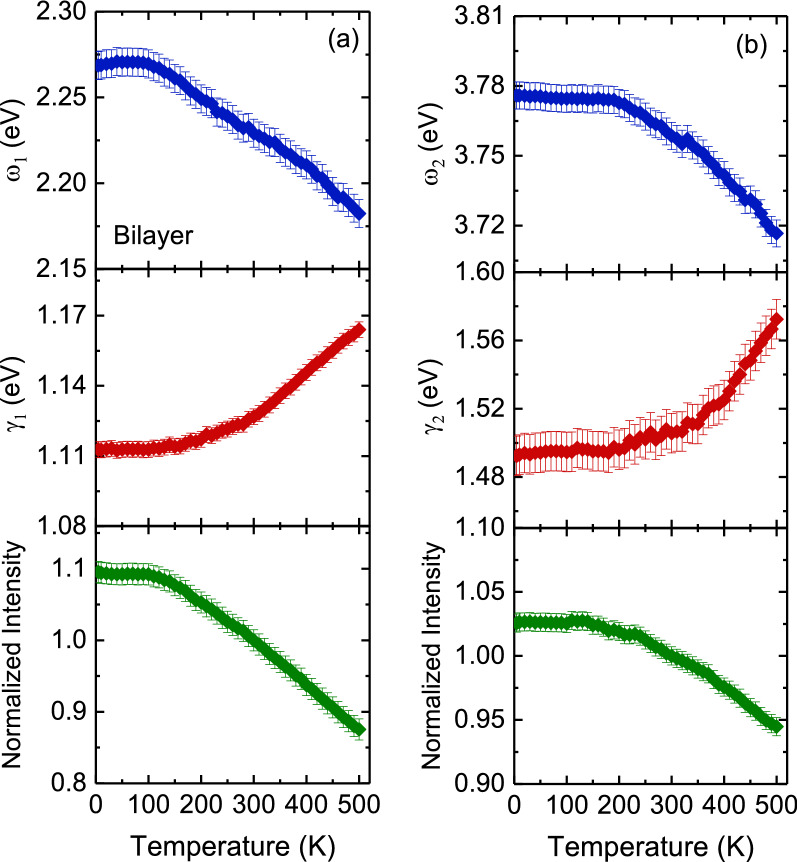
Temperature dependence of the peak energy, damping, and normalized intensity of (**a**) 2.22- and (**b**) 3.75-eV optical absorptions for bilayer PtSe_2_ thin film.

**Figure 8:**
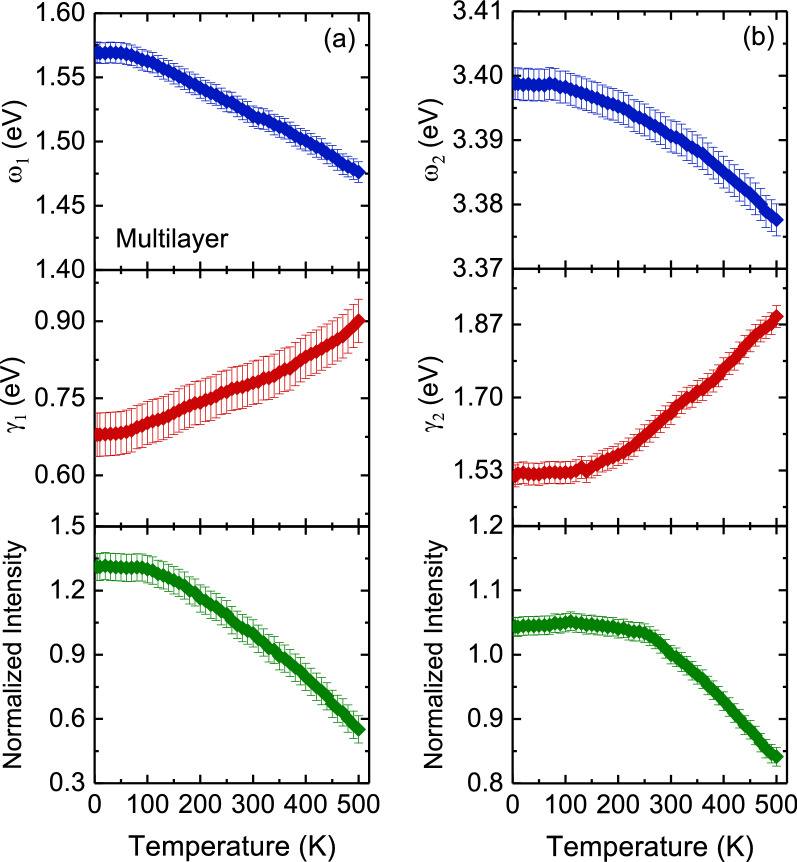
Temperature dependence of the peak energy, damping, and normalized intensity of (**a**) 1.51- and (**b**) 3.39-eV optical absorptions for multilayer PtSe_2_ thin film.

Figure 6 displays the temperature dependence of the band gap of bilayer and multilayer PtSe_2_ thin films. The band gap of bilayer PtSe_2_ thin film increased with a decrease in temperature. The observed blueshift value of the band gap energy with decreasing temperature in semiconductors can be described using the Bose–Einstein model as follows^44^:

2 $$ E_{g} \left( T \right) = E_{g} \left( 0 \right) - \frac{{2a_{B} }}{{\exp \left( {\frac{{{\Theta }_{B} }}{T}} \right) - 1}} , $$

where *E*_*g*_ (0) represents the band gap at 0 K, $a_{B}$ is the strength of the electron–phonon interactions, and ${\Theta }_{B}$ is the average phonon temperature. Our fitting results indicated that the band gap energy toward 0 K was approximately 0.83 ± 0.01 eV. The strength of the electron–phonon interactions *a*_*B*_ and the average phonon temperature *Θ*_*B*_ were 66 meV and 288 K, respectively. These values are comparable to those obtained for other semiconducting materials such as ZnS^45^ and ZnSe^46^. In contrast to bilayer PtSe_2_ thin film, multilayer PtSe_2_ thin film revealed a band gap energy that steadily increased with an increase in temperature from 310 to 500 K. This could induce semimetal to semiconducting transition and may be due to both the electron–phonon interaction and thermal expansion of the lattice^47^. The linearly fitted result indicated that the temperature coefficient was 1.75 × 10^−4^ eV/K. This value is lower than those of other TMDs such as ReS_2_^48^. Figures 7 and 8 illustrate the peak energy, damping, and normalized intensity of 2.22- and 3.75-eV optical transitions for bilayer PtSe_2_ thin film and 1.51- and 3.39-eV optical transitions for multilayer PtSe_2_ thin film as a function of temperature. The peak positions of all absorption bands shifted to higher photon energies, resonance damping narrowed, and normalized intensity increased with a decrease in temperature.

**Figure 9:**
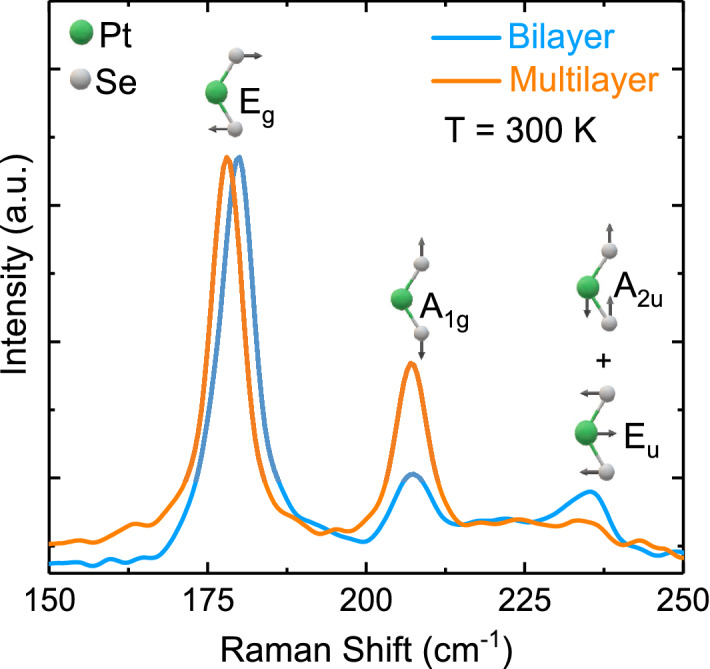
Raman scattering spectra of PtSe_2_ thin films at room temperature. The inset illustrates vibrational modes of both PtSe_2_ thin films ^50^.

Figure 9 depicts the room-temperature Raman scattering spectra of bilayer and multilayer PtSe_2_ thin films. The spectra comprised three Raman phonon modes. We fitted these phonon peaks using a standard Lorentzian profile. The phonon frequency and assignment are summarized in Table 2. According to the results of factor group analysis, PtSe_2_ had a 1* T*-type hexagonal crystal structure (*P-3m1*) with one formula unit per primitive cell^27^. The irreducible representation of the phonon modes at the center of the Brillouin zone is expressed by Γ = *A*_*1g*_ + *E*_*g*_ + 2*A*_*2u*_ + 2*E*_*u*_^27^. These modes are classified as Raman active (*A*_*1g*_ + *E*_*g*_), infrared active (2*A*_*2u*_), and Raman and infrared active (2*E*_*u*_). We observed two main peaks at approximately 179 and 207 cm^−1^ in bilayer PtSe_2_ thin film, which were associated with the zone center and first-order one-phonon emission for in-plane and out-of-plane with *E*_*g*_ and* A*_*1g*_ symmetries_*,*_ respectively. The peak frequencies (179 and 207 cm^−1^) reproduced previous Raman scattering measurements, indicating a bilayer signature^27^. Additionally, we observed one less-predominant peak at approximately 235 cm^−1^, which corresponded to the longitudinal optical (LO) mode in bilayer PtSe_2_ thin film. This mode is mainly separated into two vibrations that correspond to first-order two-phonon emissions for out-plane (*A*_*2u*_) and in-plane (*E*_*u*_) motions of Pt and Se atoms. Similar phonon modes have been observed in CdI_2_ as the same structure with PtSe_2_ thin films^49^.

**Table 2:** Raman phonon modes observed at room temperature for bilayer and multilayer PtSe_2_ thin films with their corresponding assignments.

Layer type	ω_1_ /*E*_*g*_ (cm^−1^)	ω_2_/*A*_*1g*_ (cm^−1^)	ω_3_/LO (cm^−1^)
Bilayer	179	207	235
Multilayer	178	207	232

In the normalized condition of the intensity of the *E*_*g*_ mode, two phonon modes (*E*_*g*_ and LO) of PtSe_2_ thin film shifted to lower frequency ranges (from 179 and 235 cm^−1^ [bilayer] to 178 and 232 cm^−1^ [multilayer], respectively) with an increase in the number of layers. Unlikely those two phonon modes, the frequency of the *A*_*1g*_ mode of bilayer PtSe_2_ thin film was unchanged when the number of layers increased. This anomalous behavior is consistent with that of PtSe_2_ thin film growth through molecular beam epitaxy^50^. Subsequently, we observed that the *A*_*1g*_ mode of multilayer PtSe_2_ thin film exhibited higher intensity than that of bilayer PtSe_2_ thin film because of stacking-induced structural changes and long-range Coulombic interlayer interactions^51^. The intensity of the LO mode decreased with an increase in thickness. This behavior could be associated with the enhanced interlayer coupling in multilayer PtSe_2_ thin film^27^.

**Figure 10:**
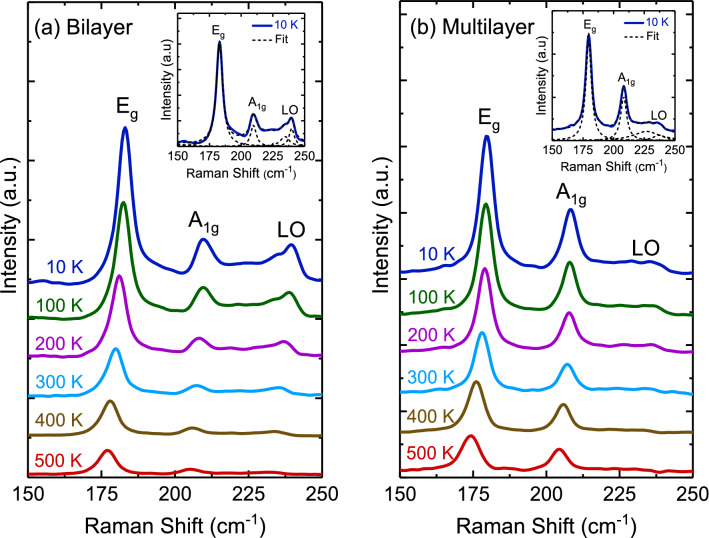
Temperature-dependent Raman scattering spectra of (**a**) bilayer and (**b**) multilayer PtSe_2_ thin films. The inset displays Raman scattering spectrum with Lorentzian oscillators of bilayer and multilayer PtSe_2_ thin films at 10 K.

**Figure 11:**
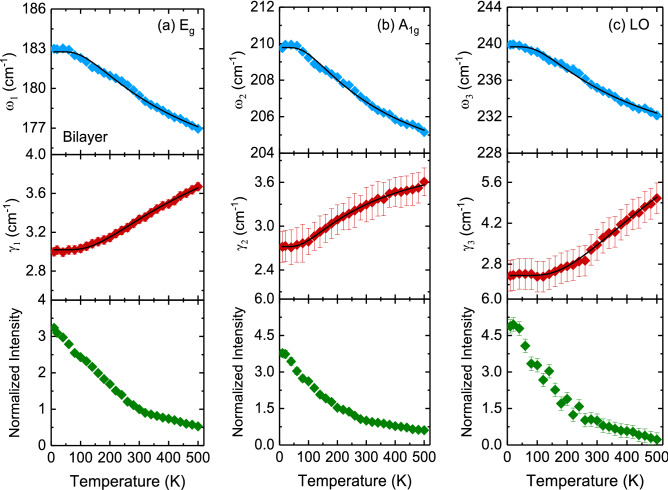
Temperature dependence of the frequency, linewidth, and normalized intensity of (**a**) *E*_*g*_, (**b**) *A*_*1g*_, and (**c**) LO modes for bilayer PtSe_2_ thin film. The thin solid lines indicate the results of the fitting from the anharmonic model by using Eqs. (3) and (4).

**Figure 12:**
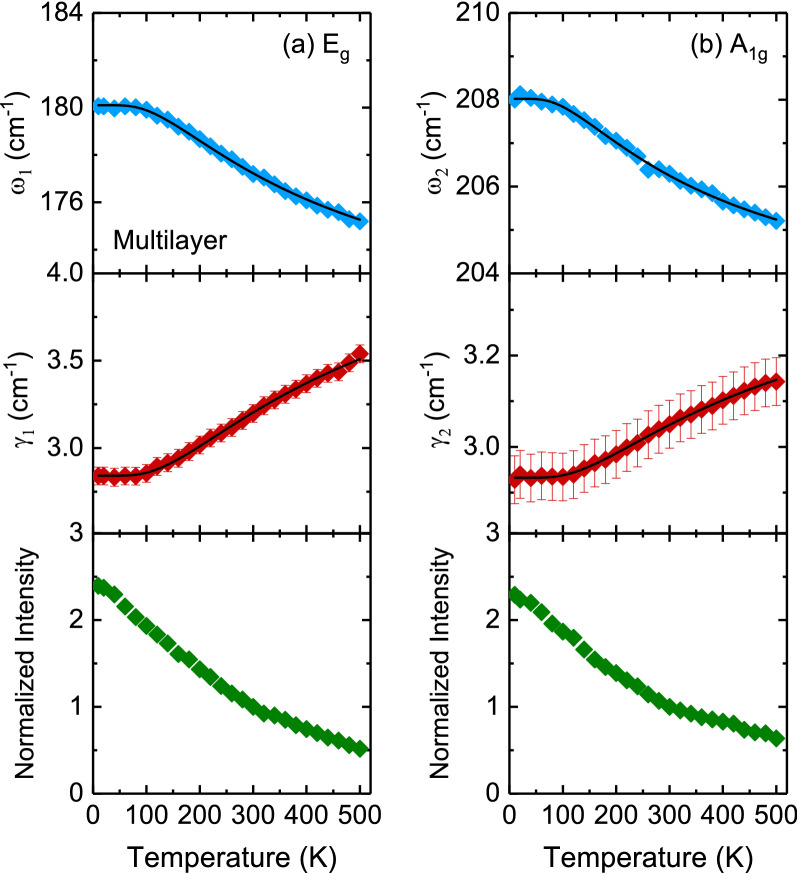
Temperature dependence of the frequency, linewidth, and normalized intensity of (**a**) *E*_*g*_ and (**b**) *A*_*1g*_ modes for multilayer PtSe_2_ thin film. The thin solid lines indicate the results of the fitting from the anharmonic model by using Eqs. (3) and (4).

Figure 10 displays the temperature dependence of the Raman scattering spectra of bilayer and multilayer PtSe_2_ thin films. When the temperature decreased, the peak positions of all phonon modes shifted to higher frequencies, and their resonance linewidth decreased. The Raman scattering spectra exhibited sharp phonon modes at 10 K for both thin films. Four Lorentzian oscillators were used to represent the Raman scattering spectrum at 10 K (inset of Fig. 10), whereas the background was taken to be linear in these fits of the form *Aω + B*, where *A* and *B* are adjustable parameters. Figures 11 and 12 illustrate the frequency, linewidth, and normalized intensity of phonon modes as functions of temperature. The phonon modes changed continuously from temperatures of 500 to 10 K. In a normal anharmonic solid, a decrease in temperature causes an increase in phonon frequency but a decrease in linewidth. Anharmonic interactions are relevant to higher-order terms of atomic vibrations that are beyond traditional harmonic terms. The temperature-dependent phonon frequency and linewidth can be expressed as follows^52^:

3 $$ \omega \left( T \right) = \omega_{0} + A\left( {1 + \frac{2}{{exp\left( {\frac{{\Theta }}{T}} \right) - 1}}} \right) $$

4 $$ \gamma \left( T \right) = \gamma_{0} + B\left( {1 + \frac{2}{{exp\left( {\frac{{\Theta }}{T}} \right) - 1}}} \right) $$

where *ω*_*0*_ is the intrinsic frequency of the optical phonon mode, γ_0_ is linewidth broadening due to defects, *A* and *B* are anharmonic coefficients, and *Θ* is the Debye temperature. The values of the fitting parameters are summarized in Table 3.

**Table 3.** Parameter values obtained from fitting the temperature dependence of phonon frequencies and linewidths according to the anharmonic model using Eqs. (3) and (4).

Layer type	Mode	*ω*_*0*_ (cm^−1^)	*γ*_*0*_ (cm^−1^)	*A* (cm^−1^)	*B* (cm^−1^)	$\Theta$(K)
Bilayer	*E*_*g*_	182.8	3.0	− 6.1	0.9	382
	*A*_*1g*_	209.8	2.6	− 4.2	0.7	313
Multilayer	*E*_*g*_	180.1	2.8	− 5.2	0.8	390
	*A*_*1g*_	208	2.9	− 2.7	0.3	339

Table 3 indicates that the high absolute *A* values of *E*_*g*_ and *A*_*1g*_ modes in bilayer and multilayer PtSe_2_ thin films revealed a dominant anharmonic interaction between Pt and Se atoms. These large values are crucial for reducing lattice thermal conductivity^53^. The relatively small absolute *B* values of *E*_*g*_ and *A*_*1g*_ modes in bilayer and multilayer PtSe_2_ thin films were associated with strong anharmonic phonon–phonon interactions. Thus, anharmonic phonon scattering with three-phonon process (Eqs. 3 and 4) is highly favorable for bilayer and multilayer PtSe_2_ thin films. At a temperature of 10 K, as depicted in Fig. 10, the LO phonon mode of bilayer PtSe_2_ thin film exhibited a peak position that split into two-phonon modes (*A*_*2u*_ and *E*_*u*_) at approximately 235 and 239 cm^−1^. By contrast, multilayer PtSe_2_ thin film exhibited a broadening peak at approximately 236 cm^−1^. Moreover, the *A*_*2u*_ and *E*_*u*_ phonon modes of bilayer PtSe_2_ thin film exhibited a blueshift and an increase in intensity in temperature with a decrease in temperature.

### Summary

The temperature-dependent optical properties of bilayer and multilayer PtSe_2_ thin films were investigated through spectroscopic ellipsometry and Raman scattering spectroscopy. Large value of the refractive index (approximately 6.88) in the near-infrared frequency range was obtained for multilayer PtSe_2_ thin film. The thermo-optic coefficients of bilayer and multilayer PtSe_2_ thin films were (4.31 ± 0.04) × 10^−4^/K and (–9.20 ± 0.03) × 10^−4^/K at a wavelength of 1200 nm. The room-temperature optical absorption spectra revealed that bilayer PtSe_2_ thin film had an indirect band gap of approximately 0.75 ± 0.01 eV, whereas multilayer PtSe_2_ thin film exhibited semimetal behavior. The band gap of bilayer PtSe_2_ thin film increased to 0.83 ± 0.01 eV at 4.5 K. By contrast, multilayer PtSe_2_ thin film exhibited a band gap energy of approximately 0.04 ± 0.004 eV at 500 K. Moreover, the temperature-dependent phonon frequency and linewidth of Raman-active *E*_*g*_ and *A*_*1g*_ modes of both thin films accorded with the predictions of the anharmonic model. These results provide fundamental information regarding PtSe_2_-based devices for optoelectronic and photonic applications at various temperatures.

## Data availability statement

The data that support the findings of this study are available from the corresponding author upon reasonable request.

## Supplementary information

Supplementary Information
