# Supplementary material for: Temperature-dependent optical and vibrational properties of PtSe2 thin films
Source: Sci Rep. 2020 Nov 4;10:19003. doi: 10.1038/s41598-020-76036-y (PMC7643157; doi:10.1038/s41598-020-76036-y)
Supplement: Supplementary file 1 — Supplementary Information [file 41598_2020_76036_MOESM1_ESM.pdf]

Supplementary information

Temperature-dependent optical and vibrational properties of $PtSe_2$ thin films

Desman P. Gulo[1], Han Yeh[2], Wen-Hao Chang[2], and Hsiang-Lin Liu[1,*]

[1]Department of Physics, National Taiwan Normal University, Taipei 11677, Taiwan
[2]Department of Electrophysics, National Chiao Tung University, Hsinchu 30010, Taiwan

*Corresponding author: hliu@ntnu.edu.tw

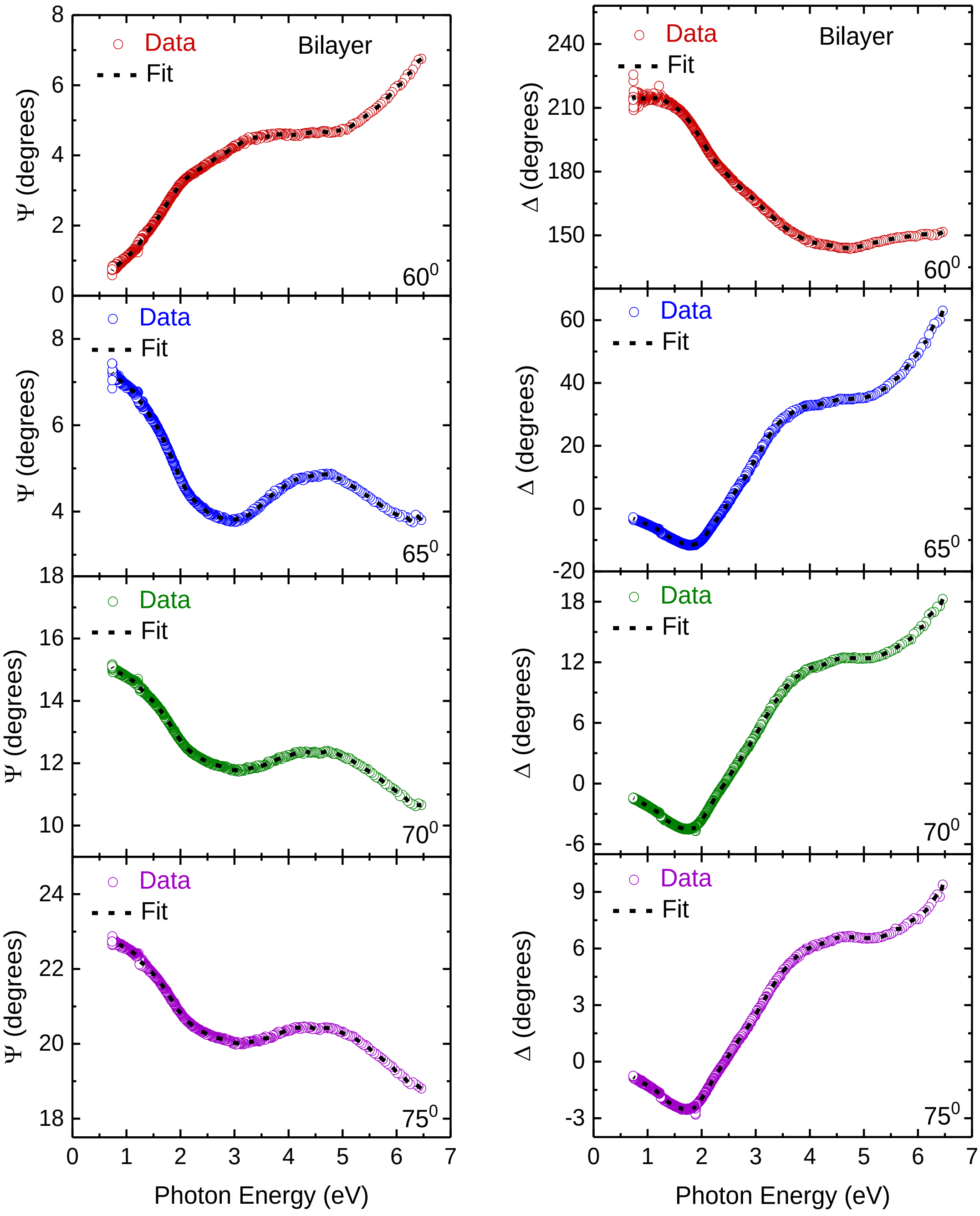

Supplementary Fig. 1. Room temperature experimental at 60º, 65º, 70º, and 75º incidence angles and fitting model of ellipsometric parameters of psi (Ψ) and delta (Δ) of bilayer $PtSe_2$ thin film.

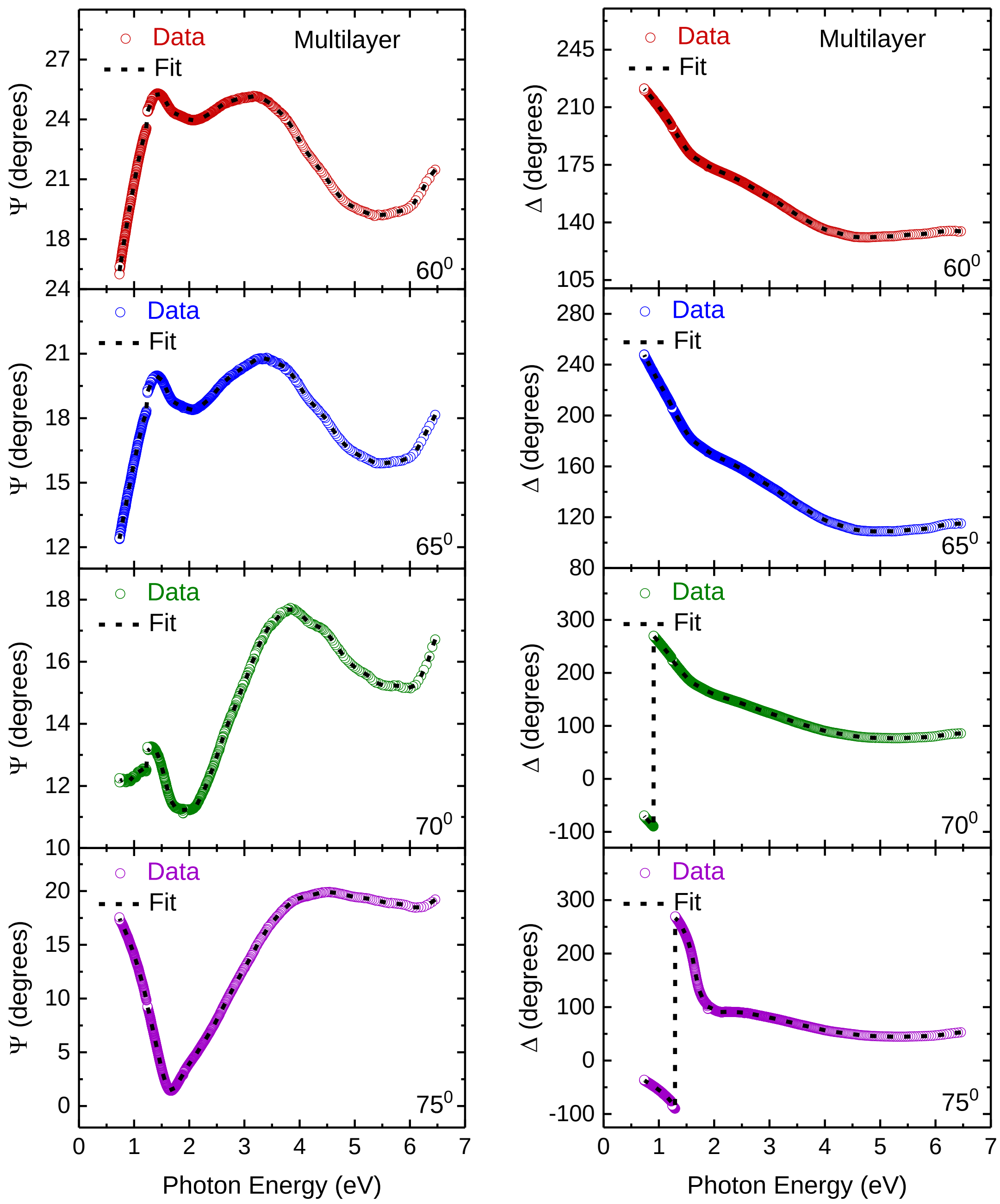

Supplementary Fig. 2. Room temperature experimental at 60°, 65°, 70°, and 75° incidence angles and fitting model of ellipsometric parameters of psi (Ψ) and delta (Δ) of multilayer $PtSe_2$ thin film.

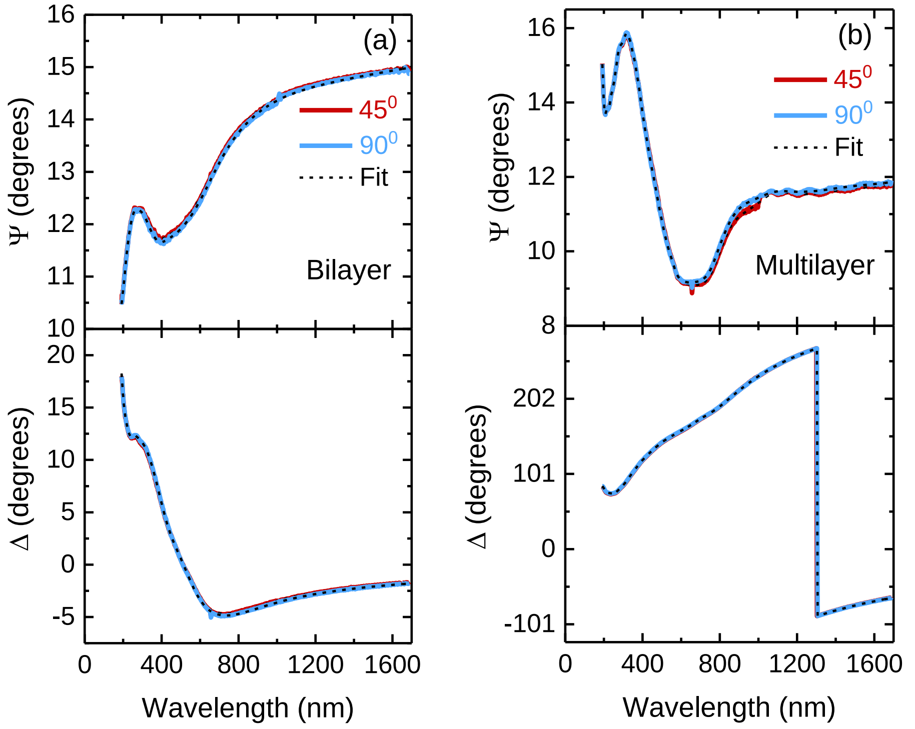

Supplementary Fig. 3. Room temperature experimental at 70° incidence angle and fitting model of ellipsometric parameters of psi (Ψ) and delta (Δ) of (a) bilayer and (b) multilayer $PtSe_2$ thin films.

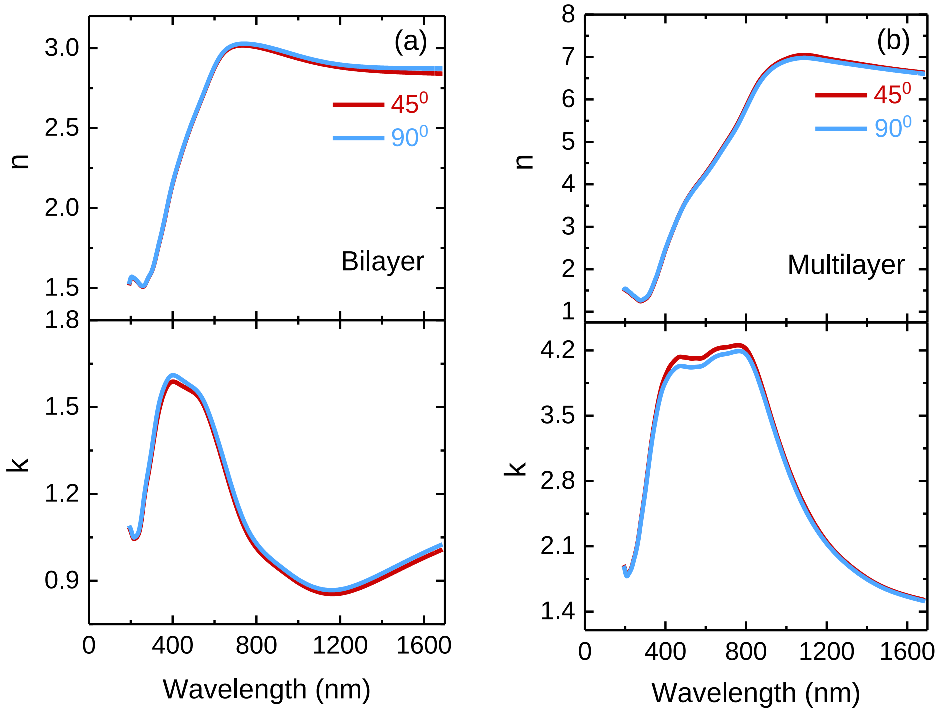

Supplementary Fig. 4. Room temperature refractive index and extinction coefficient of (a) bilayer and (b) multilayer $PtSe_2$ thin films by rotating the sample's azimuthal orientation of 45 and 90 degrees.

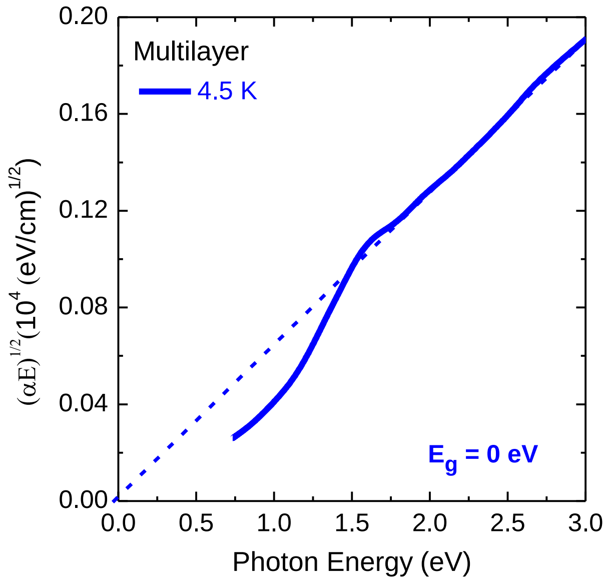

Supplementary Fig. 5. The plot of $(\alpha \cdot E)^{1/2}$ vs. photon energy enables the extraction of indirect band gap of multilayer $PtSe_2$ thin films at 4.5 K.
